# Supplementary material for: Whole-Transcriptome Analysis Reveals the Regulatory Network of Immune Response in Dapulian Pig
Source: Animals (Basel). 2024 Dec 8;14(23):3546. doi: 10.3390/ani14233546 (PMC11639921; doi:10.3390/ani14233546)

Supplementary Figure S1. Sample correlation and DEGs in Dapulian and Landrace, and immune-related genes specific to Landrace

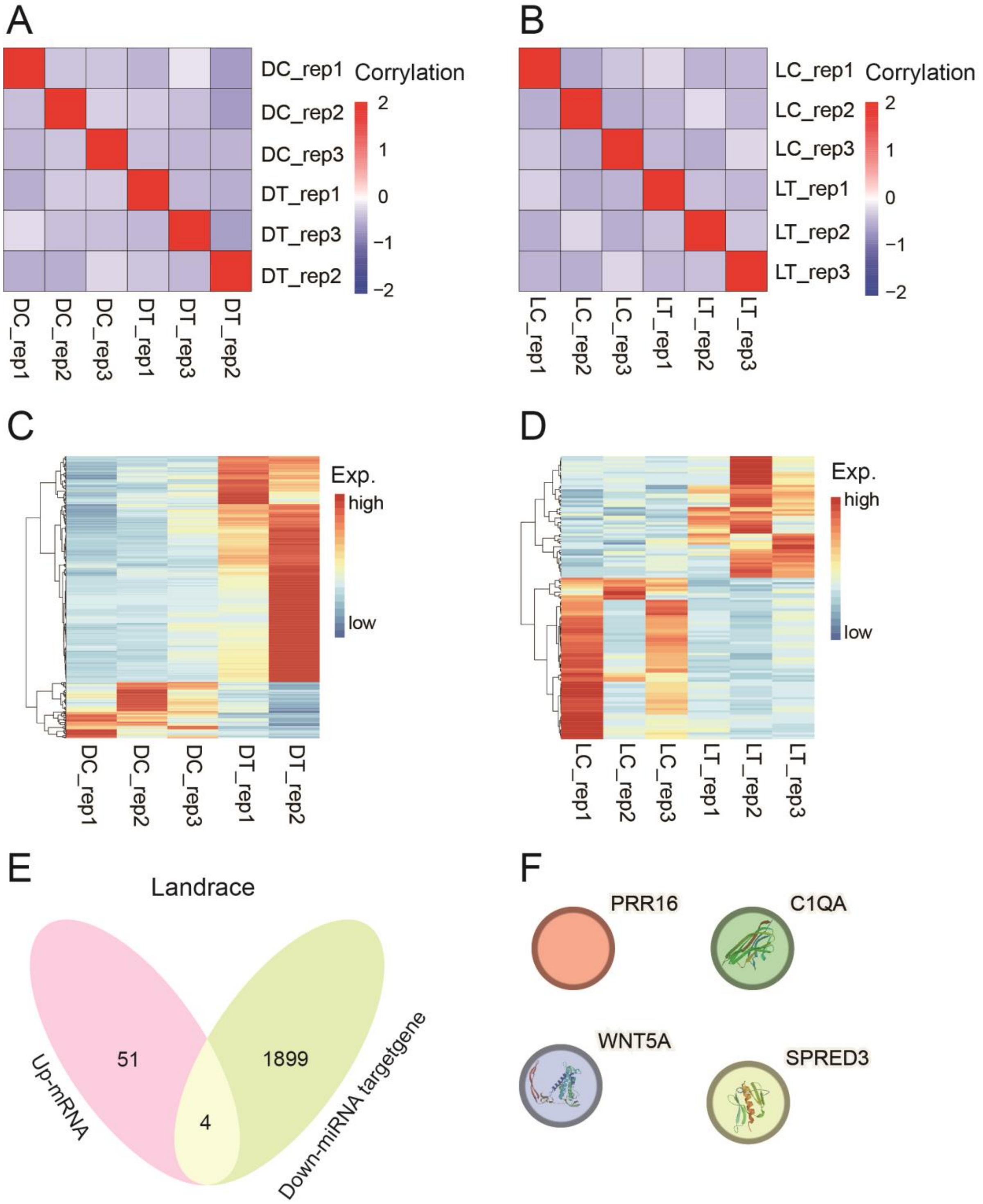

Supplement: Supplementary file 1 [file animals-14-03546-s001.zip › Figure S1.pdf]
